# Fluid-based wearable sensors: a turning point in personalized healthcare

**DOI:** 10.55730/1300-0527.3588

**Published:** 2023-05-22

**Authors:** Berfin VURAL, İnci ULUDAĞ, Bahar İNCE, Canan ÖZYURT, Funda ÖZTÜRK, Mustafa Kemal SEZGİNTÜRK

**Affiliations:** 1 Department of Bioengineering, Faculty of Engineering, Çanakkale Onsekiz Mart University, Çanakkale, Turkiye; 2 Department of Chemistry and Chemical Processing Technologies, Lapseki Vocational School, Çanakkale Onsekiz Mart University, Çanakkale, Turkiye; 3 Department of Chemistry, Faculty of Arts and Sciences, Tekirdağ Namık Kemal University, Tekirdağ, Turkiye

**Keywords:** Biosensor, tattoo sensors, healthcare monitoring, wearable sensors, biofluids

## Abstract

Nowadays, it has become very popular to develop wearable devices that can monitor biomarkers to analyze the health status of the human body more comprehensively and accurately. Wearable sensors, specially designed for home care services, show great promise with their ease of use, especially during pandemic periods. Scientists have conducted many innovative studies on new wearable sensors that can noninvasively and simultaneously monitor biochemical indicators in body fluids for disease prediction, diagnosis, and management. Using noninvasive electrochemical sensors, biomarkers can be detected in tears, saliva, perspiration, and skin interstitial fluid (ISF). In this review, biofluids used for noninvasive wearable sensor detection under four main headings, saliva, sweat, tears, and ISF-based wearable sensors, were examined in detail. This report analyzes nearly 50 recent articles from 2017 to 2023. Based on current research, this review also discusses the evolution of wearable sensors, potential implementation challenges, and future prospects.

## 1. Introduction

Until 60 years ago, electroanalytical methods were performed using large benchtop measuring instruments and large electrodes [1]. Studies using these large-sized devices have focused on customizing the electrodes by chemical immobilization to improve the analytical performance of the sensors. With the development of nanomaterials 30 years ago, the first steps were taken to miniaturize electrochemical systems [2]. With the attempt to miniaturize all systems, the use of biosensor systems has become easier, and at the same time, metabolites can be determined more quickly and cheaply. In the 2000s, there have been important developments such as solid-contact potentiometric sensors, compact handheld electrochemical instruments, microfluidic systems, and the production of flexible electrode materials [3,4]. With the development of nanotechnology, the different and superior properties of materials in nanosize have also started to be used. For example, nanomaterials such as carbon nanotube and graphene in sensors have increased [5]. These studies formed the basis of the wearable sensor technology being studied today.

Today, the term “wearable” transcends traditional clothing and represents a personalized accessory that enables simultaneous information processing [6]. With the desire to take healthcare services out of the hospital and monitor patients for long periods, the trend toward wearable sensors has begun. In addition, the use of wearable sensors to monitor various health-related biometric parameters during daily activities has recently become the focus of researchers’ attention. Wearable sensors can analyze various bodily fluids such as sweat and ISF and provide information about individuals’ biochemical statuses. Many individuals are familiar with equipment such as wearable heart rate monitors and pedometers for medical reasons or as part of a fitness program [7]. This information can be used to improve personal health through early detection of disease and paves the way for early intervention in patients.

**Figure 1:**
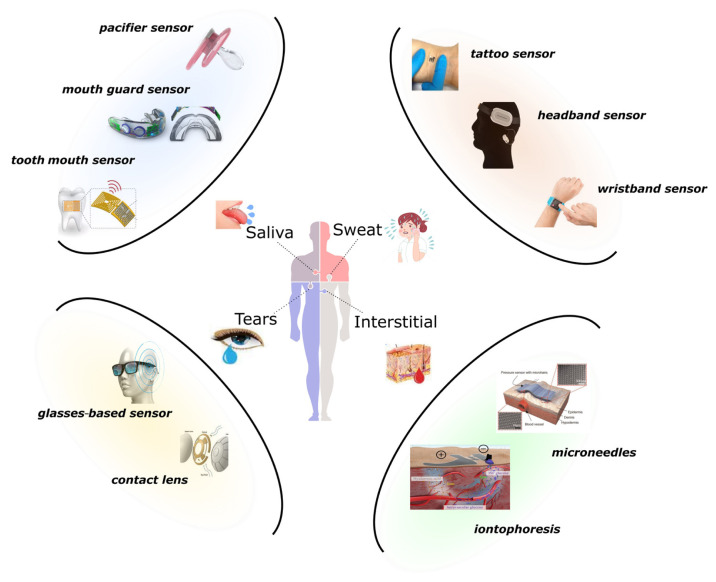
Schematic representation of tattoo biosensors.

Wearable sensors can provide real-time and continuous monitoring of health-related biosignals in a user-friendly way compared to expensive and large traditional devices [8]. Sensors to be used for health status monitoring are demanded not only to have precise and stable biosensing capability but also to be designed with optimum mechanical flexibility for a harmonious integration with the human body [9–11]. For this reason, it is of great importance that the sensing materials, the basic elements of wearable sensors, are compatible with the mechanical structure against deformations that may occur during wear. The latest studies aim to automatically upload the data collected by the sensor devices to the cloud, process and summarize it, present it to the doctor for evaluation, and finally implement the designs to give feedback to the patient [12]. This collected data and the physician evaluation complete the physical examination remotely. Thus, it becomes possible to create a comprehensive treatment plan by reducing the patient burden in hospitals and the workload of healthcare workers [13]. However, wearable chemical sensors are complicated due to many factors to overcome, such as sampling, collection, distribution, sensor calibration, wearability, and safety. In addition, despite the growing success of sensors that monitor physical properties, relatively little study has been done on wearable chemical sensors that simultaneously monitor bodily fluids such as tears, sweat, urine, and blood [14]. However, direct-to-skin goggles [15], contact lenses [16], wristbands [17], mouth guards [18], gloves [19], and tattoo sensors (Figure 1) [20] provide continuous and comfortable monitoring of human biofluids (saliva, tears, ISFs, and sweat). By lowering the need for harmful chemicals and materials, decreasing the number of samples needed for analysis, and creating sensors that need minimum or no sample preparation, wearable biosensors may also contribute to green chemistry, green analytical chemistry, and green sample preparation.

Many types of transducers, such as thermoelectric, capacitive, piezoelectric, optical, and electrochemical transducers, are used in wearable sensors [21]. Thermoelectric converters measure thermal changes, capacitive converters measure physical signals such as force, pressure, and displacement, optical converters measure photosensitive reactions, while electrochemical converters measure redox changes in chemical reactions. Each of these converters has its own advantages and disadvantages. According to our literature review, sensor designs on electrochemical and optical converters have been quite common in wearable sensor studies in recent years.

This review summarizes the breakthroughs that paved the way for modern wearable sensor systems for the noninvasive monitoring of biomarkers found in human biofluids. In this context, the benefits and disadvantages of each metabolite used in the studies were discussed in light of current studies. At the same time, the future expectations of these developments were mentioned. A comparative analysis was conducted between various review articles to present each study’s shortcomings. We hope that this review will provide convenience for researchers in future studies.

## 2. Application areas of the wearable sensors

Due to their ease of use, flexibility, and simultaneous traceability, wearable sensors are incredibly beneficial for personalized healthcare. Wearable sensors record environmental and physiological data from the human body automatically. The use of wearable sensors to detect a variety of biofluids in health, sports, and biomedical applications offers tremendous promise. Today, sensors that measure the heart rate [22], blood pressure [23], oxygen saturation [24], movements [25], respiratory rate [26], and body temperature [27] are extensively employed.Real-time monitoring systems facilitate the diagnosis and treatment of chronic diseases, especially in personalized medicine. Wearable sensors allow the health status of individuals with chronic diseases to be monitored simultaneously and continuously. They would be especially useful for the care of elderly and sick individuals and the early diagnosis of potential patient attacks. This review has focused on wearable sensors for early disease diagnosis due to their noninvasive nature, rapid results, and low cost. Specifically constructed wearable sensors for diseases like cancer, for which early intervention and treatment are crucial, will facilitate the patient’s treatment [28]. Measured data from wearable sensors can be sent to a mobile device or access point via wireless or internet connections. Thanks to these features, they can send a warning message to the response centers in an emergency. Based on online data, medical professionals can remotely monitor patients and make urgent decisions about their care if necessary.

Wearable sensors can be successfully integrated into clothing items and fashion accessories such as glasses, socks, headphones, shoes, glasses, hats, and wristbands, keeping patients’ comfort at the highest level during continuous monitoring and diagnosis. Future wearable sensors will be able to keep the user’s health under control, thereby enhancing the quality of life. In recent years, the technology market has been flooded with wearable sensors that measure health-related data and have high user acceptance. However, these devices are used in daily life almost exclusively for fitness.

## 3. Biofluids used for noninvasive wearable sensor detections

Noninvasive interventions are typically nonsurgical processes. The most common way to discover biomarkers associated with any disease is to analyze blood samples from the body. Traditional invasive diagnostic procedures like bloodletting and finger swabs can induce pain at the blood draw site. Taking a sample of perspiration, tears, saliva, breath, or sweat is painless and simpler than taking a blood specimen. This convenience promotes patient comfort. Noninvasive methods would be the preferred alternative in cases where blood sample collection is difficult, such as in newborns, the elderly, and hemophobic individuals [29]. Moreover, expertise in the implementation of intrusive procedures is required. The study of noninvasive biomarkers using wearable sensors offers the prospect of on-site diagnosis without the need for professionals and therefore has potential for individualized health monitoring. As a result, collecting the sample by an expert for biomarker detection will be less crucial. Using easily collectable fluids such as saliva and sweat instead of taking blood samples is one of the main advantages of wearable chemical sensing.

In the past five years, scientists have made great strides in constructing wearable sensors that simultaneously monitor these biological fluids without requiring invasive procedures.

### 3.1. Saliva-based wearable sensors

Saliva offers an excellent alternative to blood analysis to monitor the human body’s emotional, hormonal, nutritional, and metabolic state and has attracted the attention of many researchers to develop diagnostic tools [30]. Saliva is a mildly acidic, mucous exocrine secretion [31]. Since saliva contains many compounds that pass through the bloodstream, it is an important biofluid for noninvasive detection methods. It can potentially be a resource for monitoring an individual’s oral and general health. Important substances in saliva include minor metabolites, proteins, and hormones [32]. The contents of saliva can be used for medical and early diagnosis. Saliva contains a variety of medically significant analytes that can be used as biomarkers for various disorders, including cancer [33] and autoimmune diseases [34].

On the other hand, salivary biomarker concentrations are often lower than those seen in blood and other noninvasive biofluids. Due to the lower availability of analytes, biosensors to be designed should have lower detection limits. At the same time, food particles, bacteria, and other contaminants present in saliva samples can limit the operating capabilities of saliva-based detection platforms. Thus, a saliva sample preparation step, such as filtering or centrifugation, may be required before assessing salivary biomarkers. The diagnostic use of saliva has attracted scientists working in the field of wearable sensors, as it does not require an invasive method and it is easy to collect samples.

In saliva-based wearable sensor design, platforms such as mouth guard bases or tooth tattoos [35] have often been developed to directly access the oral cavity. The first examples of such wearable sensors were developed by Preton et al. in the 1960s [36].

Studies have shown that salivary lactate concentrations and blood lactate levels are compatible and can be used in vitro to monitor health conditions [37–39]. Using screen-printed enzymatic electrodes on a mouth guard, Wang et al. created the first wearable real-time sensor that monitors the lactate metabolite in saliva. They introduced new intraoral biosensor concepts by integrating a printable amperometric enzymatic biosensor into an easily removable mouth guard platform. The basic operation and basic functions of the sensor design are as follows: (i) first, a graphene-based sensing element with a wireless readout coil is fabricated on silk fibroin, and (ii) a nanosensor is transferred to biomaterials such as tooth enamel by dissolving the supporting silk film, (iii) graphene and the extremely large surface area of the electrodes provide high adhesion suitability to solid surfaces of biomaterials, (iv) battery-free operation, and ease of use with its remote wireless sensing feature. Mouth guards are widely used by athletes to prevent dental injuries. Unlike permanent saliva sensors, the mouth guard sensor can be easily fitted and replaced by the user without any special assistance. This saliva-based mouth guard biosensor exhibited high sensitivity, selectivity, and stability. This first mouth-guard-based biosensor designed by Wang et al. has been a pioneering work for many researchers [35]. This advanced amperometric mouth guard biosensing concept can easily be extended to saliva tracking of other clinically relevant biomolecules. Thus, it provides useful information about the user’s health and performance in various biomedical and health applications.

**Figure 2:**
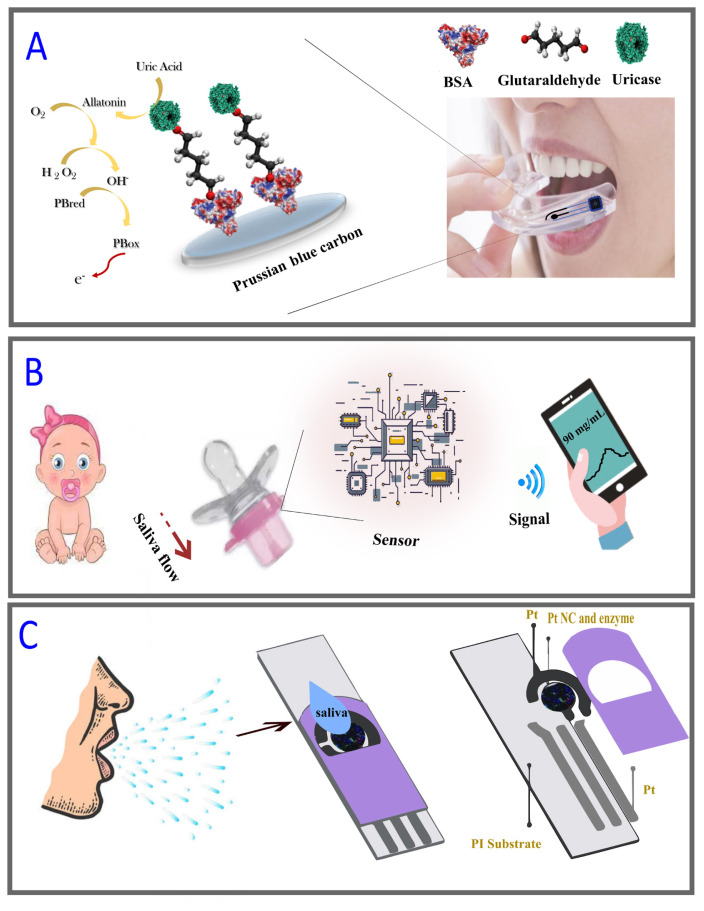
Examples of saliva-based wearable sensors. (A) Image of the mouth guard biosensor , (B) photograph of infancy biomarker detection by the wireless pacifier-based biosensor, (C) image of an enzyme-based biosensor for the determination of cholesterol in human saliva [42].

Kim et al. presented a mouth guard that can monitor salivary uric acid levels noninvasively [18]. The screen-printed electrode (SPE) system modified with Prussian Blue/Uricase/glutaraldehyde/poly-ortrophenylendiamine could detect uric acid amperometrically. The modified SPE system is integrated into a mouth guard platform with anatomically miniaturized instrumentation electronics, including a potentiostat, a microcontroller, and a bluetooth low energy transceiver (Figure 2A). A small printed circuit board has been connected to the new wearable mouth guard biosensor to enable wireless data collection. The electrochemical performance of the biosensor is attractive and demonstrates good selectivity, sensitivity, and stability.

Another similar biosensor in the mouth guard platform was recently designed by Mitsubayashi et al. [40]. The newly created biosensor is mounted on the mouth guard to monitor salivary glucose, which has been reported to correlate with blood glucose levels. The developed sensor can measure glucose in saliva without any pretreatment. Since pollutants such as ascorbic acid and uric acid prevent accurate measurement of glucose concentration in human saliva, cellulose acetate was added to the electrode as an interference barrier to limit the interference effect, and a successful response was obtained. The cellulose acetate membrane is biocompatible for human use as a mouth guard material, and polyethylene terephthalate glycol was chosen for its safety. Also, a nickel-plated conductive spring was used to connect the electrodes and the wireless electronic circuit. It has been reported that the sensor can measure glucose concentration in the range of 1.75–10,000 μmol/L.

According to studies, an abnormality in saliva indicates health problems in the oral cavity and ailments affecting the stomach, lungs, and other organs [41]. In the last five years, it is understood that there has been an increased interest in electrochemical sensors in wearable sensors designed to determine salivary biomarkers. These sensors basically consist of miniature transmitters, liquid junction-based electrodes, and a power supply. In particular, wearable electrochemical sensors printed on the mouth guard and pacifier have shown potential for continuous monitoring. Most sensor studies are generally aimed at detecting the glucose level in saliva. While pacifier sensors are preferred to provide a comfortable diagnosis in newborns, mouth guards are widely used as support material in adults.

In addition to mouth guard platforms, systems such as pacifiers [42], wristbands [43], tooth tattoos [44], and rings [45] have also been used to detect analytes in saliva samples.

Mishra et al. designed a sensor ring platform to simultaneously measure THC (Δ9-tetrahydrocannabinol) and alcohol [45]. This research aims to detect drunk drivers in traffic, fight drug trafficking, and assist on-the-spot forensic investigations. In addition, an effort was made to create a fast, reliable, easy-to-use detection tool for law enforcement. A voltammetric sensor for THC detection and an amperometric sensor for alcohol detection are included in the ring sensor. According to the obtained data, the sensor gave a stable and smooth response to alcohol concentration linearly between 0.1 mM and 0.6 mM.

The design of portable, harmless monitoring equipment is critical for newborns who are unable to express any discomfort or medical problems. Laura et al. developed the first chemical wearable sensor for newborn monitoring after observing that all devices developed for monitoring wearable babies are only capable of monitoring physical parameters (Figure 2B) [42]. In this study, saliva was evaluated as a noteworthy biological fluid for diagnostic use in infants because of its good correlation with blood sugar levels, easy and painless acquisition, and widespread availability [46]. In this system, saliva is pumped into the pacifier using this fully integrated structure as a result of mouth movements so that the target molecule enters the integrated electrochemical sensing chamber together with the saliva. The biosensor operates on a Prussian blue electrode converter based on glucose-oxidase-based enzymatic sensing. Wireless communication is essential to ensure the portability and user comfort of skin-implanted devices. An isolated electrochemical detector to prevent materials from leaking into the mouth, a compatible wireless amperometric circuit combined with Bluetooth, and a nontoxic polymeric pacifier cap are the basic components of the pacifier detection platform developed in this study.

A completely integrated electrochemical wristband sensor was released in 2022 to track the amounts of phenylalanine (PHE) in serum and saliva [43]. The wristband platform also makes it simple to quickly swap the disposable sensor strip between tests, eliminating contamination. The biosensor analyzes phenylalanine in a biofluid using a tiny potentiostat coupled to an altered SPE. In the first step, a Nafion and sodium 1,2-naphthoquinone-4-sulphonate (NQS) derivatization layer was deposited on top of the carbon SPE to enable the electrochemical detection of PHE. The analytical performance of the sensor was then thoroughly described. The developed system exhibited a dynamic range in the buffer of 20 to 1000 M, possibly providing monitoring from healthy to dangerous levels.

While cholesterol today is mostly analyzed from serum samples, researchers have recently tried to measure cholesterol in bodily fluids such as tears, sweat, and saliva. An electrochemical-based cholesterol sensor, reported by Eom and colleagues in 2020, aimed to measure the cholesterol found in saliva. Nevertheless, it is very difficult to determine the low cholesterol levels in saliva accurately. Therefore, Pt nanoclusters (Pt-NCs) were used to improve sensor performance (Figure 2C). The SPE electrode modified with Pt nanocluster/enzyme/Nafion tested a limit of detection (LOD) value as low as 2 μM. Designing simultaneous analysis of cholesterol levels in saliva and serum will provide a meaningful database for precise and accurate diagnosis in the future [47].

Table 1 provides a summary of typical wearable sensors based on saliva in recent years [18,35,40,42–45,134–137].

Although saliva sensors offer continuous real-time detection, they face several limitations, particularly the possibility of leakage of several teeth integrating the sensors. While work in the discipline of saliva-based wearable sensors is very exciting, it has the disadvantage that the composition of salivary fluid can often change with a person’s last meal. Also, temperature changes affect the response of potentiometric sensors. Therefore, the system must also include a temperature sensor to obtain reliable results.

The oral cavity is a semiclosed space that provides a highly controlled environment, especially during sleep. This concept helps oral wearable sensors to be more reliable and accurate by successfully reducing interference from the external environment. Saliva-based biosensor systems promise a great future in real-time health status monitoring by precisely optimizing system components and measurement conditions.

### 3.2. Sweat-based wearable sensors

Sweat is a liquid secreted by sweat glands that contains broad chemical information. Na^+^, Cl^−^, K^+^, Ca^2+^, pH, NH^4+^, glucose, lactate, ethanol, uric acid, ascorbic acid, Zn^2+^, Cd^2+^, Pb^2+^, Cu^2+^, Hg^+^, and cortisol are just some of the basic compounds and ions that can be found in sweat [21,48]. Human metabolites directly associated with diseases or other human health conditions are abundant in sweat. Sweat contains numerous other components, including hormones, proteins, and peptides, which are difficult to detect due to their trace amounts. For example, excessive sodium and potassium loss through sweat has been associated with hyponatremia, muscle cramps, and dehydration [49]. It has been reported that sweat glucose is related to blood glucose [50], and sweat lactate is potentially a sensitive marker of pressure ischemia [51]. In addition, sweat-associated uric acid and tyrosine are associated with metabolic diseases such as gout [52].

Due to the widespread distribution of sweat glands in the human body and many biomarkers in sweat, sweat fluid plays an important role in wearable sensor technology [21]. Studies with sweat glands have concluded that their concentration in sweat may be directly related to blood [53]. In the future, if the concentrations of these molecules in sweat can be determined, information about the body’s homeostasis mechanisms and general health status can be given. For example, considering the relationship between the presence of neuropeptide Y in sweat and depression, a sensor to be developed in the future may allow inferences about the individual’s mental health [54].

Besides their high efficiency, sweat-based wearable sensors are expected to meet several minimum requirements, including flexibility, durability, high stretchability, biocompatibility, lightness, and low power consumption. Using techniques such as prolonged exercise or pilocarpine iontophoresis to analyze sweat, it is possible to quickly obtain it and transport it to a wearable device [55,56]. Placing wearable sensors close to the site of sweat formation allows rapid detection of analytes before they biodegrade. Accessories such as wristbands [17,57], headbands [58,59], gloves [60], functional skin bands [20,61–66], bands [66], and skin pads [67] have the potential to be used for simultaneous invasive sweat monitoring. Several wearable sweat sensors have been created recently.

Jagannath et al. created a wristband sensor to detect sweat-associated cytokines, including interleukin-6, interleukin-8, interleukin-10, and tumor necrosis factor [57]. This study evaluates the infection/inflammatory response by identifying host immunological biomarkers in sweat. The “SWEATSENSER” device developed in the relevant work provided stable and real-time monitoring of inflammatory cytokines in passive sweat. The wristband sensor has been reported to show a reproducible response in the range of 2 to 200 pg/mL. Such a wearable sensor would be helpful for users to take necessary precautions before experiencing symptoms of infection.

It is not surprising that the analysis of sodium ion (Na^+^), a nontoxic component that controls the body’s electrolyte balance and water content, in sweat samples is the subject of current and future studies, considering its amount in the relevant body fluid. The concentration of sodium ions, an important indicator of many physiological disorders in the human body, is high in sweat [68]. The sweat sensor in a headband platform for sodium analysis was developed by Ghoorchian et al. [59]. Na_0.44_MnO_2_ was used as the sensing material in the designed biosensor due to its good Na^+^ incorporation ability, electrical conductivity, stability, and low fabrication cost. This biosensor aimed to detect Na^+^ ions in human sweat samples in real time. The developed sensor successfully determined the amount of Na^+^ in the linear range of 0.21–24.54 mmol/L. Although this study led to the development of wearable sensors in the future to assess Na^+^ ions, the sensing material also reacted to Ca^2+^ ions in sweat. Therefore, with the development of this study, it is expected that it will be possible to design wearable sensors with higher specificity in the future. The concept of integrating the sensor into the headband can be considered quite useful compared to similar studies. Wearable sensors can be divided into two broad classes, flexible [69] and inflexible [70]. For easy physiological data collection from sweat, fabrication materials must be flexible and robust for dynamic use on human skin [71]. The platform for wearable sweat sensors that attracted the most attention from scientists was skin patch/tattoo sensors. Many tattoo sensors have been developed, especially for glucose determination [61–64]. Bolat et al. have recently designed the state-of-the-art wearable sweat glucose sensor [62]. Researchers developed a soft and flexible wearable sweat epidermal microfluidic device that stimulates, collects, and analyzes sweat electrochemically. They created the first method to combine an iontophoretic pilocarpine delivery system around the intake channels of a sweat collection and analysis of epidermal polydimethylsiloxane microfluidic devices. The device, easily adhered to the skin, consists of an integrated system developed for stimulating sweat secretion and collecting and analyzing sweat. Freshly produced sweat differs from its counterparts in that it is naturally pumped into the fluid inlet without the need for exercise. This study aims to prevent sweat samples from leaking and reduce sample collection time by integrating epidermal iontophoretic microfluidic devices.

For some user groups, such as patients and the elderly, exercising or going into the sauna to collect sensor measurements is difficult and may even be physically impossible. In order to provide physiological insights without obstructing users’ lives, sweat sensors must be able to autonomously access sweat without harming the skin and be compatible with daily activities. Although secretion rates are typically relatively low, sweat is secreted naturally even during sedentary or routine activities such as sitting, walking, and sleeping. The fingertips, palm, and back of the hand have some of the highest sweat gland densities in the body. Therefore, they are suitable body areas for natural sweat access. Researchers have presented a new glove-based sensing platform for analyzing sweat naturally secreted on the hands and fingers during sedentary or routine activity. The sensor, designed using gold and bismuth electrodes, electrochemically determines components such as alcohol, zinc, chloride, and pH, especially on the glove surface in contact with the back of the hand, which is a region with high sweating rates. Similarly, a three-electrode system was designed in nitrile finger cots to detect vitamin C levels. The shown glove-based sensor devices offer a simple, user-friendly method of analyzing natural sweat. The developed sensors were tested on subjects and proved adaptable for physiological monitoring applications [60]. Potentiometric devices provide a relatively simple technology for sweat detection, but the balance between reference and test solutions is a major source of error. In 2017, Choi and colleagues developed a sweat-embedded chloride sensor with an integrated salt bridge that minimizes balance and ensures stable measurements over long periods [72].

**Figure 3:**
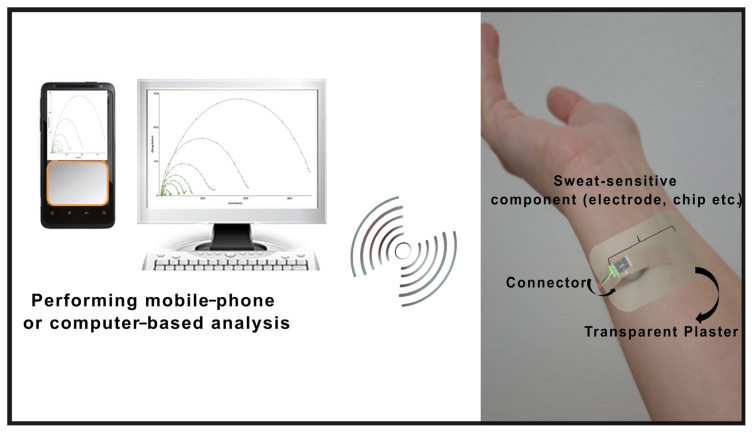
A schematic representation of sweat-based wearable sensors integrated into the forearm using a transparent adhesive bandage.

Photoelectrochemical (PEC) sensors, an advanced electrochemical method capable of higher sensitivity, have rarely been applied to designing flexible wearable sweat devices. Also, tattoo sensors have been getting a lot of attention lately because of their great promise in countless applications. In particular, research based on temporary tattoo electrodes sticking to the skin surface has become widespread (Figure 3). In 2022, a flexible skin patch consisting of an ultrasensitive PEC sensor for high-performance sweat glucose detection and a hydrophilic poly-(dimethylsiloxane) sweat collector that stimulates sweat secretion was presented by Liu et al. The flexible PEC sweat sensor comprises a three-electrode system prepared on the above-obtained patterned multiwalled nanotube (MWNT) electrode. The stability and performance of the sweat sensor were further characterized under various modes of mechanical deformation such as compression, stretch, torsion, and pressing. The proposed skin sensor offered excellent resistance to mechanical deformations, selectivity, high storage stability, and low detection limit (22.2 pM) [63].

Another tattoo-based sweat sensor developed for in situ detection of human sweat samples was made by Cui et al. The designed sensor is an optical glucose biosensor integrated into the skin pad [67]. The fact that colorimetric sensing can detect without using electrical connections provides design freedom and makes it easy for individuals to use it on their own and while traveling. Cui et al. created a platform based on glucose-sensitive proportional fluorescent nanohybrids for noninvasive and visible monitoring of sweat glucose. In this study, the skin pad, red in the absence of glucose under fluorescent light, turned blue by glucose determination in the sweat sample. The researchers aimed to examine glucose in sweat optically. This method is less sensitive but more practical than electrochemical sensors that detect glucose from sweat.

Another current wearable glucose sensor is designed using screen-printed gold electrodes (rGO-Au/SPGE) modified with graphene oxide and gold nanoparticles [73].

An ultrasmall wearable sweat biosensor system was presented by Wang et al. In the sensor, which mainly contains a printed circle board (PCB) and flexible electrodes, the MSo_2_ chip served as the key component of the wearable sensor to receive and process electrochemical data. A lithium battery powered the system. The dynamic change of four markers in sweat (glucose, lactate, Na^+^, and K^+^) was successfully recorded after an athlete put a flexible band on his forehead after cycling for 10 min [74].

One recent development in sweat-based wearable biosensors is a new multifunctional sweat-based electrochemical-physiological hybrid skin patch for simultaneous monitoring of sweat glucose, pH, temperature, and EKG, which could be functional for smart wearables. This design basically consists of two electrochemical and two physiological sensors, potentiometric pH sensor, amperometric glucose biosensor, electrocardiography (ECG) sensor, and temperature sensor, respectively. These sensors are integrated on a flexible polyimide substrate [75].

Diagnosing cortisol is important because cortisol is a hormone that plays a vital role in regulating many physiological processes in the body, including metabolism, immune response, blood pressure, and the body’s response to stress. In a 2023 study, a flexible, wearable localized surface plasmon resonance (LSPR) aptasensor was created by immobilizing the aptamer on a polydimethylsiloxane (PDMS) substrate to determine the concentration of cortisol in the human body [76].

One of the latest developments in sweat-based wearable biosensors is the use of microfluidics to control the flow and composition of sweat. Generally, the device consists of a small patch with microchannels that adheres to the skin and directs sweat to a sensor for analysis. The sensor can detect various biomarkers such as organic metabolites and drugs in sweat and transmit the data wirelessly to a smartphone or other device [77].

Thanks to the good accuracy and high sensitivity of electrochemical sensors, they have been used in many studies for sweat sensors (Table 2) [17,20,57,59–67,72–76,138–142].

Current wearable sweat sensors use various techniques to find important analytes, including colorimetry, voltammetry, amperometry, and potentiometry. Wearable potentiometric ion sensors have played a leading role among these devices in monitoring athletic performance and medical treatment.

While blood fluid analysis can directly detect certain diseases, the relationship between sweat analyte levels and health status is still not fully known. Elucidating the mechanisms associated with the blood level of sweat analytes will increase the ability of sweat-based wearable sensors to provide more reliable results and improve their use in daily life. Obtaining a large enough sample volume for analyte detection is similarly difficult. Various methods have been developed between sweat collection and testing, considering the limitations of sensor design, such as evaporation, chemical degradation, and mixing old sweat with new sweat [62,72]. Nevertheless, more progress needs to be made on its real-life applicability.

Good adhesion to moving surfaces is a priority when the sweat sensor is attached to human skin. It should be considered that the surface will be subject to lateral tension, compression, and bending at the joints. Under these conditions, the sensor must have high flexibility to adhere to the body. Because without the stretch feature, the sensor attached to the skin is likely to separate from the surface. In this case, the reliability of the sensor may wane. Although sweat sensors have sufficient flexibility and good adhesion, they can experience wear and tear over time if exposed to repeated and prolonged mechanical stresses. The developed sensor must be capable of self-healing, as this significantly limits its long-term durability [9].

Sweating is a byproduct of heat exposure or strenuous activity. However, the nature of sweating caused by heat or exertion may not be an accurate reflection of a person’s normal state of health. Therefore, procedures that induce local sweat secretion may be preferable to obtain reliable results [21,55].

As a result, it is still a challenge to design wearable sweat sensors. The main difficulty is that parameters vary depending on the person and environment. The calibration problem, skin residues, or environmental factors can contaminate sweat until it reaches the working electrode. Moreover, they are not reusable, and real-time measurement is difficult due to their disposable design [78]. If these limitations are completely overcome, more sensitive and reliable wearable sweat sensors can be available in the future.

### 3.3. Tear-based wearable sensors

Tear fluid is an attractive biofluid for wearable sensor technology, as it is less complex than blood, contains many biomolecules, and does not require invasive intervention for sample collection. Many biomarker molecules pass directly from the blood to the tears. pH, Na^+^, Cl, K^+^, Mg^2+^, Ca^2+^, urea, HCO_3_, pyruvate, ascorbate, glucose, cytokines, protein, dopamine, lactate, and cortisol are among the many health-related signal molecules found in tears [79,80]. Concentrations of biomarkers in blood and tears show a strong relationship. It is also thought that the development of tear-based analyses will enable the detection of ocular diseases [69]. Tears contain more than 20 different biomolecules, including glucose, proteins and various metabolites [70,81,82]. The tear constituents respond to differences in ocular and systemic metabolic states. Consequently, they can be used to determine the physiological condition of a person [81].

It has been reported that tears produced reflexively against external stimuli have different components from basal tears, which form the protective tear film covering the eye surface. Therefore, the demand to design a wearable biosensor that does not cause eye irritation has been highlighted [83]. Contact lens-based platforms solve tear sample collection problems as they do not irritate the eye and come into direct contact with basal tears [69]. However, the biocompatibility of contact lenses can be an obstacle.

**Figure 4:**
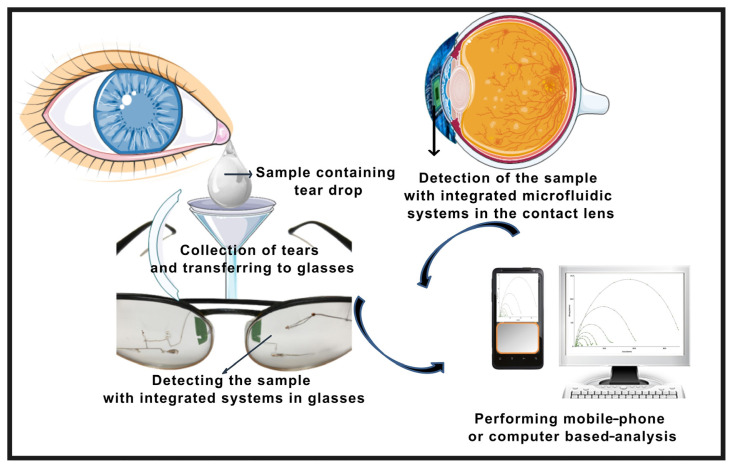
Schematic representation of the basic working principle of the systems developed for the analysis of tear samples.

In tear-based wearable sensor designs, scientists have generally focused on glucose determination [16,84–89]. Since the mechanism of glucose sensors is well known, the features of the platforms have been improved with studies. For this reason, the tear-based glucose sensors led to the development of noninvasive detection platforms to determine more physiologically important biomarkers. Optical and electrochemical biosensing are two types of contact lens-based sensors in widespread use (Figure 4) [90].

Because they are comfortable for the wearer, consistently produce tear fluid, have high oxygen permeability, and enable precise continuous monitoring, contact lens devices make an attractive platform for fabricating tear-based sensors. Several key biomarkers, including hydrogen ion (pH), protein, ascorbic acid, and glucose, can be measured in human tears simultaneously using noninvasive wearable biosensors [91]. Moreddu et al. developed microfluidic contact lenses as wearable platforms for in situ measurements of tear pH, glucose, protein, and nitrite ions [87]. With CO_2_ laser ablation, the planned microfluidic system was printed on commercial contact lenses. The developed platform consists of a central ring with 4 branches, and the designed biosensors are integrated into the microcavities at the ends of these branches. Artificial tears were used to test the contact lens sensor, which was then read utilizing a smartphone-MATLAB algorithm interface. As a result of the characterization studies, the pH sensor has a sensitivity of 12.23 nm/pH and a LOD of 0.25 pH units. On the other hand, glucose, protein, and nitrite sensors have a sensitivity of 0.49 nm/g/L, 0.49 nm/g/L, and 24.43 μmoL, respectively. This study is very promising for simultaneous monitoring of different analytes.

The electrochemical biosensing technology in contact lens-based sensors is a significant development. Kajisa et al. created an extremely sensitive hydrogel field effect transistor (FET) glucose sensor electrode that may function as a wearable gadget based on tears. The signal noise carried on by nonspecific adsorption may be considerably reduced by the sensor electrode [92].

Zhou et al. proposed a simple and rapid process for synthesizing a new composite material prepared of bimetallic oxides of Fe and Co and reduced graphene oxide (Fe_x_Co_y_O_4_-rGO). The addition of Fe resulted in the creation of a composite with a uniform distribution structure. The nonenzyme glucose detection platform fabricated by drop-casting materials onto flexible carbon electrodes produced from Fe_x_Co_y_O_4_-rGO demonstrated outstanding glucose detection capability with a detection limit as low as 0.07 M. It was utilized to dynamically assess the glucose content of tears and is anticipated to be implemented in healthcare and diagnostic equipment [93]. Researchers have recently become interested in flexible FETs that are mechanically flexible for wearable applications because of their quick reaction and simple-stable structure. Huang et al. proposed a flexible and transparency GFET-based graphene-based wearable nanosensor. The nanosensor is successfully prepared with a receptor that can attach selectively to the biomarker, resulting in a measurable change in the graphene carrier concentration. L-cysteine, linked to several disorders, was selected as the biomarker to illustrate the sensor’s detecting capacity. The nanosensor can detect L-cysteine consistently and reliably with a detection limit of 0.022 × 10^−6^ M in undiluted human sweat and 0.043 × 10^−6^ M in artificial tears. The sensor is made from a 1 M polyethylene glycol terephthalate substrate and a WO_3_/Au/WO_3_ electrode, and it may be placed on the eyeball for tear detection without affecting sight. These findings suggest that the ultraflexible and transparent GFET wearable nanosensor may be useful for medical detection applications [94].

Another recent study is the development of wearable colorimetric contact lens biosensors using cerium oxide nanoparticles (CNP) [86]. Park et al. proposed the noninvasive detection of tear glucose by designing contact lenses containing CNPs in this study. They chemically conjugated CNPs with Gox using polyethylene glycol to the contact lens. As a result of glucose oxidase oxidizing glucose to peroxide, colorless Ce^3+^ is reduced to yellow Ce^4+^. The glucose concentration is proportional to the color shift. A smartphone with an image processing program can assess this color change for glucose levels. It has been reported that CNP-PEG-Gox-loaded contact lenses are not toxic to human corneal cells and endothelial cells. Lenses designed after characterization studies were tested in the eyes of diabetic rabbits, and tear glucose concentrations were measured. Surprisingly, the lenses also successfully detect glucose in human tear samples and determine the diabetes status of individuals. These findings suggest that CNP-PEG-Gox-loaded contact lenses could be used in conjunction with cell phones to monitor human tear glucose noninvasively. Deng et al. created soft and transparent smart contact lenses that could detect glucose in tear fluid with a high level of sensitivity. A sensitive glucose fluorescence probe and a reference fluorescent dye were immobilized inside the hydrogel network of the contact lenses to create the intelligent contact lenses. The luminous color of the smart contact lenses shifted from pink to blue when glucose levels increased. Smartphones could take fluorescent photos and convert them into RGB (red, green, blue) signals to quantify glucose levels. By using a smartphone, these intelligent contact lenses were able to detect glucose levels in tears between 23 μM and 1.0 mM [95].

In 2019, Sempionatto et al. created a noninvasive wearable tear alcohol biosensor fitted on glasses for the first time [89]. In this study, a teardrop bioelectronics platform is integrated into the nose bridge pad of an eyeglass. To eliminate the disadvantages that may occur due to the direct contact of the contact lens with the eye, a platform has been developed that is placed outside the eye area. It has been shown that the designed tear-based wearable sensor allows simultaneous monitoring of tear glucose, vitamins B6, B2, and C, and alcohol. In addition, positive responses were received in the study, and the measurements could be repeated.

A contribution to the recently developed work of glasses-based wearable sensors was also made by Kalasin et al. [96]. Researchers have selected small, portable, low-cost, and lightweight laboratory glasses for measuring tear creatinine. For creatinine detection, it is designed with a Cu-BDC MOF/GO-Cu(II)/Cu_2_O NP composition (a copper-containing benzenedicarboxylate metal–organic framework bound with graphene oxide-Cu(II) and hybridized with Cu_2_O nanoparticles). For the selectivity study, ten measurements were obtained in the presence of dopamine, urea, and uric acid. As a result of this research, the biosensor achieved a selectivity efficiency of 95.1 percent for measuring tear creatinine concentrations between 1.6 and 2400 M. It has been reported that kidney disorders can be monitored, and their progress can be monitored by using the recommended sensor-based glasses.

Table 3 below contains various tear-based designs for wearable biosensors [16,84–89,91,93–96].

Most tear-based wearable sensors are still in the prototype stage, and there are no commercial products available to the public for now. In particular, the difficulty in sample collection and the biochemically unsatisfactory sensing performance limit further development of tear-based wearable sensors.

Wearable contact lens tear tracking platforms have significant advantages over glasses-based biosensing systems. Since contact lenses do not cause eye irritation, they provide a basal tear sample, and there is no sample evaporation. However, new strategies need to be developed to improve the biocompatibility of contact lens platform biosensors. In addition, for the designed biosensors to be used in daily life, the relationship between tear and blood biomolecule concentrations should be revealed, and the chemistry of tears should be better understood. The development of multifunctional devices that target two or more analytes would increase the power of diagnosis and monitoring because most of the present systems concentrate on single analytes. In addition, because the eye is a delicate and valuable organ, lens-based diagnostic platforms must undergo rigorous clinical validation before the market release.

### 3.4. Interstitial fluid-based wearable sensors

ISF is a peripheral biofluid with a composition that is extremely similar to that of the blood. It can provide various data on minimally invasive or noninvasive chemical determination-based information. Small molecules such as salts, ethanol, glucose, and proteins make up the structure of ISF. These small analyte molecules are transported to the ISF by blood capillaries and diffuse into the cells to supply oxygen and nutrition. The concentrations of these minor biomarkers transferred are well correlated between ISF and blood [97]. Compared to blood, the delayed clotting of ISF gives a major benefit in analysis. Therefore, ISF may be employed for minimally invasive monitoring of biomarkers and metabolites [92,96–98]. In addition, because of the closeness of the ISF with surrounding cells, it is feasible to receive localized information on the metabolic environment of tissues [99]. Also, specific biomarkers in ISF have been documented in greater quantities than in blood [100–102]. For these reasons, ISF can be employed as an appealing alternative for noninvasive or minimally invasive monitoring of diabetes indicators. Noninvasive ISF sampling can be easily performed on the epidermis by IP and ultrasound (sonophoresis) [103,104].

ISF-based detection platforms have used reverse iontophoresis (RI) [105] or microneedles [106] to provide continuous access to ISF and real-time measurements of target biomarkers. In both these approaches, the goal is to access the ISF. In the RI approach, a gentle current is applied through the skin to induce an ion migration to remove the ISF molecules. The flow of positively charged sodium ions causes an electroosmotic flow towards the cathode since the skin’s inherent charge is negative. As a result, neutral molecules like glucose gravitate to the same electrode [21]. In microneedle sensing devices, on the other hand, the outer layer of the skin is disrupted to reach the ISF with negligible slight damage. The microneedle provides a wearable, minimally intrusive method of detecting ISF biomarkers [107]. Recently, analytes such as glucose [65,108–113], ketone bodies [110], lactate [110], apomorphine (APO) [114], alcohol [115], and urea [116] have been detected with ISF-based wearable sensor applications.

One of the microneedle-based biosensing technologies is “off-patch” detection, which includes preextraction of ISF using MNs, in vitro separation, and release of biomarkers for detection. Small amounts of ISF extracted with this method may be diluted by significant quantities of isolate, or the chemical characteristics of biomarkers may be altered during the separation process. “On-the-patch” detection on the skin is an alternative method. According to the conclusions of the studies, the “on-the-patch” detection method is the most promising [117]. However, because of limited sensitivity, stability issues, and high interference effects in in vivo detection, detection methodologies are unable to satisfy current ISF diagnostic requirements. Consequently, the development of “on the patch” detection technology is quite valuable. Mei et al. have created ultrasensitive surface-enhanced Raman scattering microneedles (IS-SERS-MNs) that detect bacterial metabolites in ISF as novel infection diagnostic biomarkers. IS-SERS-MNs detect pyocyanin, a bacterial metabolite present only in mouse dermal ISF, both directly and indirectly via diffusion into the dermis. This work demonstrates that IS-SERS-MNs are a powerful strategy for expanding the applicability of SERS-based MNs [118]. More recently, the same research group used SERS tags labeled MNs patch technique to lead the monitoring of acute peritonitis progression. Densely deposited core-satellite gold nanoparticles and 3-mercaptophenylboronic acid were used to detect H_2_O_2_, an indicator of peritonitis development, with high sensitivity and selectivity. This study provides a new way and technique for early diagnosis of acute peritonitis and evaluation of drug therapy effects [119]. Park et al. proposed a stretchable MN-mounted nanogap sensing that can be inserted into skin tissue, rapidly absorb ISF, and evaluate biomarkers in situ by magnifying the detection signals through redox cycling in nanogap electrodes. Parkinson’s disease medicine levodopa was detected by the MN-nanogap sensor at concentrations as low as 100 nM in an aqueous solution and 1 M in both the skin-mimicking gelatin phantom and porcine skin [120].

Traditional techniques for collecting ISF include suction blistering, microdialysis, open-flow microperfusion, and reverse iontophoresis. In developing a noninvasive biosensor to monitor glucose in ISF, ISF extraction via classical RI is challenging due to its low extraction flow and consistency. Yang et al. aimed to improve the limited extraction flow and low sensitivity of traditional MNs, which prevents early illness diagnosis. To achieve this, they utilized a combination of RI and MNs for the fast extraction and detection of Epstein-Barr virus cell-free DNA, a crucial biomarker of nasopharyngeal cancer. Due to this combination dual extraction effect, the wearable platform was able to isolate the cell-free DNA target from ISF with a maximum efficiency of 95.4% in as little as 10 min [121]. Cheng and colleagues developed a touch biosensor to monitor glucose in the ISF. This touch screen glucose sensor is built by combining a solid microneedle array (MA), RI sampling unit, and a glucose electrochemical sensing unit. MA can penetrate the skin painlessly and create uniform microchannels. The detection principle consists of first penetration into the skin with solid MA, followed by ISF glucose extraction via microchannels created using RI, and detection of the extracted glucose by amperometry. An improved RI sampling efficiency and consistency of ISF glucose have been reported due to the presence of microchannels [108]. Compared to RI extraction alone, the glucose extraction flow was increased by approximately 1.6 times in both in vitro and in vivo conditions with this developed biosensor. The designed biosensor was tested in diabetic and healthy rats with in vivo experiments. The results of the trials revealed that the results measured using the reported wearable platform and the findings of commercial blood glucose meters were well correlated. This smartphone-based biosensor is compact, low-cost, and simple, making it a viable application for in-home diabetes monitoring. Moreover, nonprofessional patients can utilize it.

Several electrochemical techniques for ISF extraction and in situ detection have been created [111, 122]. Bolella et al. reported the development of a microneedle (MN)-based biosensor made of highly porous gold (h-PG) for minimally invasive monitoring of glucose in artificial ISF [111]. Due to the unique shape of the MNs, the electrodeposition of h-PG is likely enhanced, resulting in a greater electroactive surface area. The gold surface of the MNs was changed by the drop-casting immobilization of 6-(ferrocenyl)hexanethiol (FcSH) as a redox mediator, followed by the drop-casting immobilization of a flavin adenine dinucleotide glucose dehydrogenase (FAD-GDH) enzyme. The developed biosensor has an expanded linear range (0.1–10 mM) for glucose detection in artificial ISF. The extremely porous layer multiplied the active surface area of the MN electrode by a factor of 100 compared to the usual bare electrode, hence increasing its sensitivity. The relevant study is the first one where an h-PG-modified MN-based electrode has been described in the literature. However, the considerable mechanical difference between solid and hollow hard MNs (Young’s modulus in the GPa range) and soft tissues (Young modulus in the MPa range) functioning as electrodes inhibits signal transmission at the interface and induces an inflammatory response. Recently, MN patches of polymeric material have been developed to extract ISF [123, 124]. Zheng et al. developed a silk fibroin-based MN patch with wire electrodes implanted in the MNs to detect glucose in the skin or plant ISF in situ. The wettable, nondissolvable fibroin hydrogel has strong mechanical strength for tissue penetration. After implantation, fibroin MNs expand and extract biomarker-containing ISF into their polymeric matrix to reach the implanted electrodes, creating a bioelectronic interface with good biocompatibility and mechanical compatibility for high-fidelity recording. This minimally invasive MN technique will aid chronic illness monitoring and smart agriculture [125].Recent research indicates that when a magnetic field is provided locally in combination with an electric field, it creates a Lorentz force that transfers ISF to the surface of the skin. This is referred to as magnetohydrodynamic (MHD) extraction. MHD extraction can collect ISF, similar to reverse iontophoresis. MHD extraction provides a number of benefits over reverse iontophoresis, including reduced applied electric current and fast extraction durations. These variables may increase the ISF sample’s representativeness and accelerate its analysis. Kemp et al. used glucose as the analyte to illustrate the diagnostic utility of MHD extraction. As a contact between the electrodes and human skin, the hydrogel was employed as a solid electrolyte. Pig skin glucose was effectively detected with an apparent sensitivity of 0.8 mA/mol (Mcm^2^). This is the first study to establish a safe and successful approach for noninvasive glucose extraction via the skin utilizing MHD in conjunction with chronoamperometric analysis [126].

MN sensors are gaining increasing attention due to their ability to access the dermal ISF and obtain useful molecular information regarding disease markers and metabolites. In diabetes follow-up, MN-based ISF glucose sensing has been shown as a minimally invasive alternative to existing CGM devices, whereas no trend has been seen in continuous monitoring of ketone bodies in ISF. In this regard, Teymuryan et al. created a technology for continuous and simultaneous monitoring of ketone bodies, glucose, and lactate indicators [110]. The technique is based on electrochemical monitoring of ketone formation, which is the most common biomarker of β-hydroxybutyrate (HB). This study presents the first use of a real-time continuous ketone body tracking (CKM) MN platform. The developed CKM MN sensor exhibited stunning analytical performance with high sensitivity (low detection limit, 50 μM), high selectivity in the presence of potential interference, and good stability during long-term operation in artificial ISF.

Efforts to include many chemical sensors into a single device are influencing the present trend of sensor integration towards a combination of varied sensor modalities. Combining multiple sensor modalities in a single wearable device can result in significant advantages. Monitoring the physiological impacts of daily activities requires wearable devices simultaneously monitoring metabolic and hemodynamic parameters. For this purpose, Sempionatto and colleagues developed a suitable, stretchy, and integrated wearable sensor capable of measuring blood pressure, heart rate, glucose, lactate, caffeine, and alcohol levels for dynamic and complete self-monitoring of health [112]. The solvent brazing procedure based on SEBs (styrene-ethylene-butylene-styrene block copolymer) delivered accurate mechanical behavior and continuous measurements of epidermal blood pressure and biomarker signaling. By spatially isolating the acoustic and electrochemical transducers and employing solid-state sensing hydrogel layers, signal crosstalk was prevented. By integrating more sensing characteristics, it is envisioned that a fully integrated, multilayer wearable health monitoring system would be created that provides insights into an individual’s health and physiological status for chronic illness prevention and treatment.

Another investigation that allows simultaneous monitoring of multiple different biomarkers in a single device was performed by Kim et al. [65]. The researchers developed the first dual epidermal fluid sampling and sensing device, fully integrated into a single, compatible wearable platform. The concept of the developed sensor has been successfully implemented through ISF removal at a cathode and sweat stimulation at an anode. This innovation allows for the simultaneous monitoring of two different biomarkers in two different epidermal biofluids in a single apparatus. Measurements of sweat-alcohol and ISF-glucose in human subjects ingesting food and alcoholic beverages were used to illustrate the functionality of the developed wearable device. This innovative wearable sensor has shown good correlations with commercial blood glucose meters and breath analyzers for noninvasive glucose and alcohol monitoring in healthy human subjects after meals and alcoholic beverage intake.

Goud et al. conducted a study to identify the APO analyte [114]. A wearable electrochemical sensor patch based on MNs is presented in this article for continuous monitoring of APO, a commonly used therapeutic medication in Parkinson’s treatment. Considering the necessity of continuous monitoring of drugs used in Parkinson’s treatment, the results of this study are very promising for Parkinson’s patients. The created sensor has been tested in the presence of possibly interfering chemicals and has shown selectivity against APO. The sensor exhibited an attractive linear response for APO with a sensitivity of 45 nA/μM (R^2^ = 0.998). The designed MN detection patch showed good analytical performance using artificial ISF and phantom gel in skin-like environments. This well-designed trial involved a significant step toward developing individualized medicine doses for effective Parkinson’s disease therapy and long-term APO monitoring.

Table 4 provides a summary of typical ISF-based sensors and their uses [65,108–113,115–126,143–146].Biologically important markers have been successfully detected, often using minimally invasive methods such as ISF-based wearable sensors. The high correlation between analytes in the blood and ISF suggests that ISF can be used to determine more complex analytes in tomorrow’s healthcare systems. On the other hand, the development of noninvasive biosensors for monitoring critical analytes in ISF is still a challenge. Such obstacles are predicted to be solved since ISF extraction through RI is limited by its low extraction flow and uniformity. It is anticipated that wearable ISF sensors will be utilized more frequently in the future with the improvements to be made in existing methods.

## 4. Concluding remarks, challenges, and future perspectives

Wearable sensor technology has advanced significantly over the last three decades, including chemically modified electrodes, miniaturized materials, printed planar electrodes, flexible materials, and lab-on-a-chip sensing [127]. Sensing testing may progressively transfer from the bench to the house and skin thanks to advances in wearable sensor technologies and the digital revolution. The progress in wearable sensing technologies paves the way for individuals to perform personal health check-ups on their own easily.

Nowadays, health services are usually provided to patients admitted to the hospital due to certain symptoms. After the doctor’s examination, the appropriate medicine for the person’s disease is given, and passive monitoring is done. The disease may progress until the patient notices the symptoms. Wearable sensor technology provides instant tracking of individuals’ health information. Early detection can be achieved if wearable sensors become reliable and widespread.

Although blood is the most widely used biofluid in medical diagnosis, using a vein or finger prick to draw blood is not a comfortable diagnostic method for patients. For this reason, chemical sensors designed to target biofluids such as tears, saliva, urine, sweat, and ISF are of interest because they are painless. Analyzing tear and saliva samples can be relatively difficult for users of these biofluids. For this reason, sweat and ISF sensors are frequently preferred for long-term use and comfort. In addition, body fluids such as sweat and tears limit the development of wearable sensors due to the interaction of low-concentration biomarkers with evaporation and contamination.

Due to the complexity of human biofluids and some mechanisms that are not fully resolved, the sensing performance of biochemical sensors is not always ideal in real-time monitoring. In order to overcome this situation, researchers working on wearable sensors must first have in-depth knowledge of the biochemical mechanisms of human beings. Thus, great success can be achieved in quickly identifying interference signals and improving sensor performance.

Over the past five years, extensive research has offered noninvasive, real-time measurement of analyte concentrations in saliva, sweat, and tears. A single mechanically flexible multiplexed system with signal processing and wireless data transfer may now incorporate many sensors thanks to manufacturing processes and materials science advancements.

Electrochemical sensing, which detects currents or potentials at functionalized electrodes to provide analyte concentrations, is the most powerful sensing approach in wearable sensor applications [128]. It is a fact that electrochemical biosensor approaches, optical biosensor techniques, and other methods are considered very advantageous in certain applications. However, wearable sensors designed to date have been relatively less successful in evaluating analytes in biofluids [129]. Colorimetric sensing using reagents that change color when the target is exposed to analytes is another common sensing approach in wearable sensor applications [130]. While these approaches have so far only been used to detect simple ions and metabolites, they have the potential to be combined with affinity-based aptamers or synthetic polymers to allow selective detection of more complex compounds in the future [131].

Some challenges limit the use of noninvasive, wearable chemical sensors in daily life. Biocompatibility, stability, and toxicity of the utilized compounds are some obstacles encountered when constructing the sensor. For instance, the sensor’s inability to give accurate answers due to mechanical deformations during regular body motions is an issue. In addition, chemical and mechanical deformation that may result from washing sensor platforms on textile materials should be considered. Due to the severity of the issues, extensive research and computation are necessary to create sensors that will not fail for an extended period and can deliver dependable responses. Some target analytes, such as proteins, may require bioaffinity methods. Continuous monitoring of these analytes demands the regeneration of receptors. Therefore, it is challenging to utilize such analyte-detecting wearable sensors regularly. Additionally, it is challenging to employ daily wearable sensors designed for heat-degradable analytes such as proteins.

One of the greatest challenges is keeping the chemicals utilized in creating the wearable sensor away from the sensor itself. In saliva-based systems, the biofilm layer that is likely to be present on the tooth might impair the performance of sensors immobilized on the plate platform. Additionally, the relation between the composition of saliva and the last food consumed should be evaluated. Additionally, contaminants from skin rashes are a potential issue. Although contact lenses have received a great deal of attention in tear-based systems, the toxicity of immobilization compounds is a significant concern. In addition, the association between analytes in these biofluids employed in wearable sensors and blood analytes is currently being explored, and additional information is required.

Miniaturizing sensors will enhance the functionality and usability of wearable devices. Miniature electrodes, which are widely utilized nowadays, are therefore crucial to wearable electronics. Miniaturized sensors must be designed in collaboration with nanotechnology technologies. In biomedical, health, and sports applications, wearable sensors are an exciting innovation for continuously monitoring the human body. It is anticipated that thanks to individualized wearable sensors, the health issues of the elderly and sick may be handled remotely, cheaply, and without the need to visit the hospital. Thus, it will significantly advance patient monitoring and treatment.

Soon, noninvasive drugs and nutrients are expected to meet the demands of personalized care and mindful nutrition [132]. In addition, it is thought that the simultaneous detection of multiple biomarkers can be achieved with the developed MN sensor arrays.

The manufacturing process of flexible sensors must be cost-effective and scalable, allowing large-area and easy production. Selecting suitable materials and methods is crucial to producing sensors with flexible strain properties. Many polymers are used as flexible support materials in wearable sensor design due to their flexibility, human friendliness, stretchability, and durability. Nanowires, nanoparticles, rubbers, carbon nanotubes, and graphene are the most widely used polymers as flexible support materials for soft strain sensors [133].

Skin-attachable and wearable strain sensors must be highly stretchable, flexible, and extremely sensitive to mimic complex and large human skin or clothing deformations and detect small skin stretches caused by blood flow or respiration [133]. However, developing strain sensors with both high stretchability and high sensitivity continues to be quite difficult, particularly for applications in customized healthcare where delicate, very sensitive sensors are needed. Therefore, new material approaches and micro-/nanostructure designs are required to enable high-sensitivity sensing for flexible sensors. Another important issue is that body-worn sensors can be properly attached to the human body. Poor adhesion can cause the sensors to slide off the skin, resulting in noise in the sensor’s response and poor signal detection. To solve this problem, strain sensors can be securely attached to human skin with the help of microfeather adhesive structures and intermediate adhesive layers.

The most important criterion in wearable sensors is their high biocompatibility, as they interact closely with humans. However, even if a skin sensor is biocompatible with human skin, prolonged adherence to the electronic device that contains the skin can cause inflammation because prolonged use prevents the skin from breathing. Therefore, during the design of sweat sensors, attention should be paid to selecting materials that will allow the skin to breathe.

As a result, wearable sensors have the potential for widespread use in various fields, such as health, environmental monitoring, and fitness. However, there are still some challenges for their commercialization and marketing. First, construction costs remain high, limiting their accessibility to a wider audience. In addition, these sensors’ accuracy and reliability must be improved to meet the standards required for medical applications. In addition, there is a need for standardization in the industry, as there are currently no universal regulations for wearable electrochemical sensors. This lack of standardization can lead to inconsistencies in the quality and performance of these sensors. Finally, effective marketing strategies should be developed to educate the public and raise awareness about the benefits of using these sensors. While wearable electrochemical sensors have great potential, addressing these challenges will be crucial for their successful commercialization and widespread adoption. Key challenges to designing wearable sensors and using them in everyday life are expected to be addressed through multidisciplinary efforts among chemists, biologists, bioengineers, biomedical engineers, electrical engineers, materials engineers, and data scientists. We foresee that the sensors designed due to this collaborative approach can be used frequently in daily life, and a complete skin laboratory can be created.

## Figures and Tables

**Table 1:** A summary of typical wearable sensors based on saliva.

Biofluid	Analyte	Type of biosensor	Platform	Specificity or LOD	References
Saliva	Uric acid	Electrochemical	Mouthguard	0–1 mM	[18]
Saliva	*Staphylococcus aureus*	Electrochemical	Graphene/silk sensor on the tooth	10^3^–10^8^ CFU/mL	[35]
Saliva	Glucose	Electrochemical	Mouth guard	1.75–10,000 μM	[40]
Saliva	Glucose	Electrochemical	Pacifier	0.1–1.4 mM	[42]
Saliva	Phenylalanine	Electrochemical	Wristband	20–100 mM	[43]
Saliva	Glucose	Dielectric	Trilayer structure on the tooth	-	[44]
Saliva	Δ_9_-tetrahydrocannabinol and alcohol	Electrochemical	Ring	0.1–0.6 mM (for alcohol), 0.1–0.4 μM (for THC)	[45]
Saliva	Glucose and nitrite	Microfluidic paper-based devices	Paper	27 μM and7 μM	[134]
Saliva	Sodium	Electrochemical	Film	5 × 10^−^^5^ to 4.27 × 10^−^^5^ M	[135]
Saliva	pH (H^+^)	Electrochemical	pH indicator	4–9	[136]
Saliva	Lactate	Electrochemical	Mouth guard	0.1–1.0 mM	[137]

**Table 2:** A summary of typical wearable sensors based on sweat.

Biofluid	Analyte	Type of biosensor	Platform	Specificity or LOD	References
Sweat	Glucose, sodium, and lactate	Electrochemical	Wristband and headband	Glucose : 0–200 μM Na^+^ :10–160 mMLactate: 30 mM	[17]
Sweat	Alcohol	Electrochemical	Tattoo	0–36 mM	[20]
Sweat	Cytokines (interleukin-6, interleukin-8, interleukin-10, and tumor necrosis factor-a)	Electrochemical	Wristband	0.2–200 pg/mL	[57]
Sweat	Sodium	Electrochemical	Headband	0.21–24.54 mmol/L	[59]
Sweat	Alcohol and vitamin C	Electrochemical	Glove	0–300 μM (for vitC)	[60]
Sweat	Glucose	Electrochemical	Tattoo	10 μM–1 mM	[61]
Sweat	Glucose	Electrochemical	Tattoo	**-**	[62]
Sweat	Glucose	PEC	Tattoo	0.1 nM to 1 mM	[63]
Sweat	Cl^−^, pH, glucose, and levodopa	Electrochemical	Tattoo	Cl: 25–200 mM Glucose: 90 mg/dL pH: 4–8 Levodopa: 0–50 μM	[64]
Sweat and ISF	Glucose and alcohol	Electrochemical	Tattoo	**-**	[65]
Sweat	pH, Na^+^, K^+^, and Cl^−^	Electrochemical	Tattoo	-	[66]
Sweat	Glucose	Optical	Skin patch	0–15 mM	[67]
Sweat	Chloride	Electrochemical	Wearable patch	10,000–150,000 μM	[72]
Sweat	Glucose	Electrochemical	Wearable patch	1.25–850 μM	[73]
Sweat	Glucose, lactate, Na+, and K+	Electrochemical	Flexible adhesive patch	Glucose: 0–300 μM Laktate: 5–25 mM, Na +: 5–160 mM K + : 1–32 mM	[74]
Sweat	Glucose, pH	Electrochemical	Skin patch	Glucose: 0.2–1 mM pH: 4–6	[75]
Sweat	Cortisol	LSPR	Wearable patch	0.1–1000 nM	[76]
Sweat	Cl–, pH, glucose, and calcium	Optical	Superwettable band	pH: 6.5–7.0 Cl: 100 mM	[138]
Sweat	pH	Optical	Skin patch	4–9	[139]
Sweat	Lactate	Electrochemical	Wearable patch	3–100 mM	[140]
Sweat	Urea	Electrochemical	Flexible adhesive patch	5–200 mM	[141]
Sweat	Na^+^ and K^+^	Electrochemical	wearable fabric	-	[142]

**Table 3:** A summary of typical wearable sensors based on tear.

Biofluid	Analyte	Type of biosensor	Platform	Specificity or LOD	References
Tear	Glucose	Optical	Contact lens	0–50 mM	[16]
Tear	Lactoferrin and Glucose	Electrochemical	Inverse opal carbon rod electrode	Lactoferrin: 0.1–5 mg/mL	[84]
Tear	Glucose	Electrochemical	Contact lens	0.4 mM	[85]
Tear	Glucose	Optical	Contact lens	0.1 mM	[86]
Tear	pH, glucose, protein, and nitrate ions	Optical	Contact lens	0.25 pH units Glucose: 1.84 mM Protein: 0.63 g Nitrate ions: 24.43 μmol	[87]
Tear	Glucose	Electrochemical	Contact lens	0.1–0.6 mM	[88]
Tear	Glucose, alcohol, and vitamins (B2, B6, and C)	Electrochemical	Eyeglasses	B2: 300 μM B6: 500 Mm C: 1000 μM	[89]
Tear	pH, protein, ascorbic acid, and glucose	Optical	The eye patch	Protein: 0.17 g/L, Glucose: 7.0 μM Ascorbic acid: 3.0 μM	[91]
Tear	Glucose	Electrochemical	Fe_x_Co_y_O_4_-rGO Flexible electrode	0.07 μM	[93]
Tear	L-cysteine	Field-effect transistor (FET)	WO3/Au/WO3 electrode	0.043 × 10^−^^6^ M	[94]
Tear	Glucose	Optical	Contact lens	23 μM to 1.0 mM	[95]
Tear	Creatinine	Electrochemical	Eyeglasses	1.6–2400 μM	[96]

**Table 4:** A summary of typical wearable sensors based on ISF.

Biofluid	Analyte	Type of biosensor	Platform	Specificity or LOD	References
ISF and sweat	Glucose (in ISF)	Electrochemical	Tattoo	Glucose: 0–160 μM	[65]
ISF	Glucose	Electrochemical	MN	0.92 mM	[108]
ISF	Glucose	Electrochemical	Graphene-based platform	0.006–0.7 mM	[109]
ISF	β-hydroxybutyrate	Electrochemical	MN	Lac: 1–10 mM HB: 0.0–1.0 mM Glucose:1–10 mM	[110]
ISF	Lactate and glucose	Electrochemical	MN	0.1–10 mM	[111]
ISF	Blood pressure, heart rate, glucose, lactate, caffeine, and alcohol	Acoustic and electrochemical	Tattoo	-	[112]
ISF	Glucose (in ISF)	Electrochemical	Tattoo	0.06 μM	[113]
ISF	Alcohol	Electrochemical	MN	0–80 mM	[115]
ISF	Urea	Electrochemical	MN	2.8 μM	[116]
ISF	Sodium	Field-effect transistor (FET	MN	2.78 × 10^−^^6^ M	[117]
ISF	Pyocyanin	SERS	MN	30 μM	[118]
ISF	H_2_O_2_	SERS	MN	1 μM	[119]
ISF	Levodopa	Electrochemical	MN	100 nM	[120]
ISF	Epstein-Barr virus cell-free DNA	Electrochemical	MN	1.1 copies/μL	[121]
ISF	Catecholamine	Electrochemical	MN	100 nM	[122]
ISF	pH	Electrochemical	A high-density polymeric MN array-based (PMNA) sensing patch	-	[123]
ISF	microRNA	Optical	MN	miRNA-141: 14 pM miRNA-155: 6 pM	[124]
ISF	Glucose	Electrochemical	A silk-MN patch	3–18 mM	[125]
ISF	Glucose	Electrochemical	A screen-printed layer of Prussian Blue that was coated with a layer containing Gox	0.2–5 μM	[126]
ISF	Apomorphine	Electrochemical	MN	0.6–0.75 μM	[143]
ISF	Lithium	Electrochemical	Conductive cotton fibers	6.3 × 10^−^^5^ to 6.3 × 10^−^^2^ ^2^ M	[144]
ISF	Glucose	Electrochemical	Microelectrodes	0–32 mM	[145]
ISF	Glucose, lactate, and alcohol	Electrochemical	MN array	Glucose : 0–40 mM Lactate : 0–28 mM Alcohol: 0–100 mM	[146]
